# Fruitarian Diet and Blood Glucose Control in Type 1 Diabetes: A Case Report

**DOI:** 10.3389/fnut.2022.752832

**Published:** 2022-02-22

**Authors:** Claudia Vetrani, Lutgarda Bozzetto, Giuseppe Della Pepa, Angela Albarosa Rivellese, Giovanni Annuzzi

**Affiliations:** Department of Clinical Medicine and Surgery, Federico II University, Naples, Italy

**Keywords:** type 1 diabetes, eating habits, fruitarian diet, glucose control, insulin pump, continuous glucose monitoring

## Abstract

Diet is a key determinant of blood glucose control in individuals with type 1 diabetes. Although dietary education is part of their clinical follow-up, many patients show a propensity to self-treatment, adopting dietary changes, often extreme, that do not consider the overall impact on health. Here, we describe the case of a patient with type 1 diabetes who switched to a fruitarian diet because of ideological beliefs. A 25-year-old man with type 1 diabetes on an insulin pump and continuous glucose monitoring on optimal blood glucose control (HbA1c 6.5%, 48 mmol/mol; glucose time-in-range 70–180 mg/dl, TIR, 90%; coefficient of variation, CV, 36%) switched to a fruitarian diet because of ideological beliefs. After 3 months on this diet, blood glucose control was still optimal (TIR 88%, CV 33%), while plasma triglycerides and liver enzymes were above normal values. After 3 more months, blood glucose control had worsened (TIR 72%, CV 37%), plasma triglyceride and liver enzymes were within normal values, and hyperkalemia was detected. In this case report, a strict fruitarian diet was associated with early negative changes in some biochemical parameters, also in presence of optimal blood glucose control. Dietary counseling remains essential in the follow-up of patients with type 1 diabetes to ensure personalized medical nutrition therapy and monitor dietary changes that may affect health but with no major impact on blood glucose control.

## Introduction

Diet is a key determinant of blood glucose control in individuals with type 1 diabetes (1). Over the years, many dietary patterns have been proposed to address high glucose variability. A plant-based diet (2) and a low-carbohydrate diet (3) are becoming popular among individuals with diabetes mainly aiming at limiting hyperglycemia. According to international nutritional guidelines (1, 4), there is no ideal distribution of calories among carbohydrates, proteins, and fats for individuals with diabetes. Therefore, in clinical practice, each patient is advised to improve overall diet quality, considering his/her eating habits, food preferences, as much as possible, and metabolic targets. However, many patients show a propensity to self-treatment, adopting dietary changes, often extreme, that do not consider the overall impact on health. Here, we describe the case of a type 1 diabetes patient who switched to a fruitarian diet because of ideological beliefs. He used social media to increase his knowledge and obtain a meal plan.

## Case Presentation

A 25-year-old man was diagnosed with type 1 diabetes at the age of 9. He is on an insulin pump and wears a sensor for continuous glucose monitoring (CGM).

**Figure 1:**
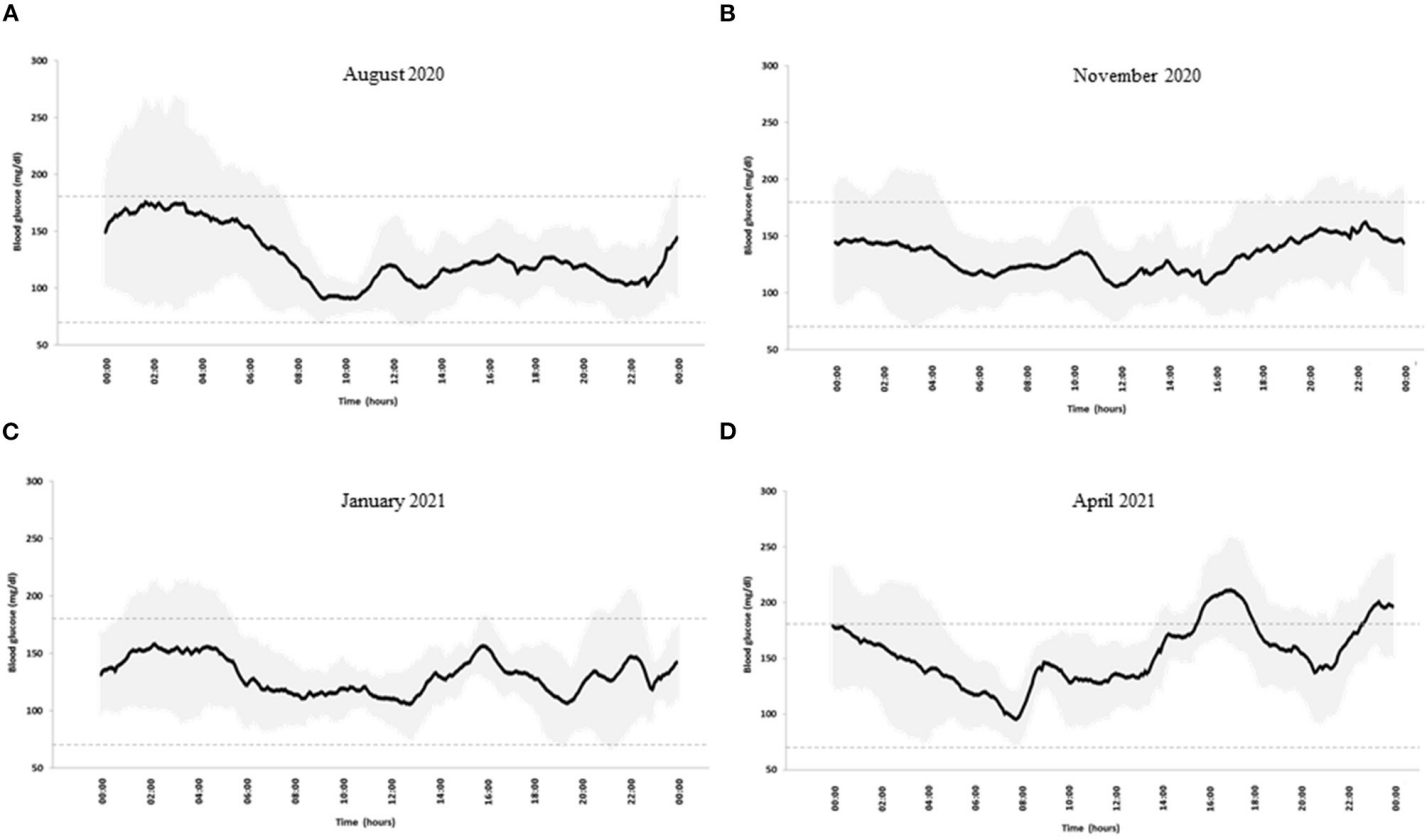
Blood glucose profile in **(A)** August 2020, **(B)** November2020, **(C)** January 2021, and **(D)** April 2021. Black line: mean blood glucose concentrations; gray shadows: glucose variability expressed as SD. Dotted lines indicate blood glucose time in the range 70–180 mg/dl.

At a follow-up out-patient visit in August 2020, his body mass index (BMI) was 24.8 kg/m^2^ and his biochemical parameters were unremarkable (Table 1). Total daily insulin doses were 24.8 ± 3.1 IU (44% bolus and 56% basal infusion). He had an optimal blood glucose control, as shown by an HbA1c level of 6.5% (48 mmol/mol) and the glucose metrics derived by CGM (5) that also showed high daily glucose variability (coefficient of variation, CV, 36%) (Table 1). A diabetologist discussed the CGM profile (Figure 1A) with the patient and did not suggest changes in insulin therapy. Dietary habits, as evaluated by a 7-day food record, mainly showed a low intake of carbohydrates and a high intake of total fats and saturated fatty acids (Table 2). In accordance with the Standards of Medical Care by the American Diabetes Association (1), a dietitian advised him to reduce the intake of saturated fatty acids and increase carbohydrates from low glycemic index foods.

**Table 1:** Anthropometric, blood glucose control, and biochemical parameters.

	**August 2020**	**November 2020**	**January 2021**	**April 2021**
BMI (kg/m^2^)	24.8	24.2	24.2	24.3
HbA1c (%)	6.5	-	6.2	6.6
HbA1c (mmol/mol)	48	-	44	49
Time in range (% blood glucose 70–180 mg/dl)	90	89	88	72
Time above range (% blood glucose >180 mg/dl)	8	9	10	26
Time below range (% blood glucose <70 mg/dl)	2	2	2	2
Mean glucose (mg/dl)	127 ± 46	127 ± 42	131 ± 44	153 ± 57
Coefficient of variation (%)	36	33	33	37
Total cholesterol (mg/dl)	142	-	125	151
LDL-cholesterol (mg/dl)	71	-	47	70
Triglyceride (mg/dl)	105	-	165	136
AST (U/l)	28	-	37	29
ALT (U/l)	22	-	45	28
GGT (U/l)	15	-	30	24

*HbA1c, glycated hemoglobin; AST, aspartate transaminase; ALT, alanine transaminase; GGT, γ-glutamyl transferase*.

**Table 2:** Mean daily energy intake and dietary composition obtained through 7-day food records during follow-up.

	**August 2020**	**November 2020**	**April 2021**	**Absolute change November ***vs***. August**	**Absolute change April ***vs***. August**
Energy (kcal)	1,977	2,030	1,980	+53	+3
Protein (g)	113.4	34.0	46.0	−79	−67
(% TEI)	24.1	7.0	9.8	−17	−14
-Animal (g)	76.8	0	0	−77	−77
(% TEI)	67.7	0	0	−68	−68
-Plant (g)	35.2	34.0	42.4	−1	+7
(% TEI)	31.0	6.7	8.6	−24	−22
Carbohydrate (g)	167.4	412.0	433.8	+245	+266
(% TEI)	33.4	79.4	86.4	+46	+53
-Starch (g)	115.3	18.0	24.0	−97	−91
(% TEI)	23.3	3.5	4.8	−20	−19
-Sugar (g)	32.6	392.5	398.7	+360	+366
(% TEI)	6.5	95.3	91.9	+89	+85
-Fructose (g)	1.59	125.3	64.5	+124	+63
Fat (g)	88.9	29.3	8.5	−60	−80
(% TEI)	42.6	13.6	4.1	−29	−39
-SFA (g)	19.5	3.8	1.8	−16	−18
-MUFA (g)	52.5	21.5	1.0	−31	−52
-PUFA (g)	12.5	2.7	2.5	−10	−10
Cholesterol (mg)	248.0	0	0	−248	−248
Fiber (g)	29.5	42.0	49.2	+13	+20
Glycemic index (%)	66.2	49.8	47.1	−16	−19
Glycemic load	98.1	155.2	156.1	+57	+58
Sodium (mg)	1,738	67.2	93.0	−1,671	−1,645
Potassium (g)	3.59	6.92	9.62	+3.3	+6.03
Calcium (mg)	738.8	269.3	420.5	−470	−318
Phosphorus (mg)	1,617	524.4	1,038	−1,093	−579
Iron (mg)	32.2	10.3	19.5	−22	−13
Vitamin C (mg)	124.6	275.8	875	+151	+750
Vitamin E (mg)	15.8	9.95	5.64	−6	−10
Vitamin D (μg)	6.98	0	0	−7	−7
Vitamin B12 (μg)	76.4	0	0	−76	−76

*MUFAs, monounsaturated fatty acids; PUFAs, polyunsaturated fatty acids; SFAs, saturated fatty acids; TEI, total energy intake*.

In November 2020, he joined an educational group to reinforce carbohydrate counting in accordance with our standard diabetes care for patients on an insulin pump. On this occasion, he told the dietitian that he had become a fruitarian in October 2020, experiencing, in his opinion, an improvement in blood glucose control. A 7-day food record confirmed that, according to the fruitarian diet, he had increased carbohydrates, mostly sugars and fructose, and fiber intake, while reducing the intake of proteins and fats (Table 2). CGM metrics confirmed an optimal blood glucose control (Table 1). Seven-day mean daily glucose variability was slightly lower (CV 33%), and the CGM profile was smoother than the previous one (Figure 1B). Total insulin doses were unchanged (24.8 ± 3.1 IU) with a shift to a relatively higher proportion of pre-prandial insulin (66% bolus and 34% basal). He reported no changes in body weight and physical activity.

In January 2021, after 3 months on the fruitarian diet, biochemical laboratory analyses showed a 50% increase in plasma triglyceride and doubling of liver enzymes. A liver ultrasound examination showed no signs of steatosis. As compared to November 2020, no changes in glucose variability, CGM metrics and profile, total insulin doses, body weight, and physical activity were observed (Table 1 and Figure 1C). Against the advice of health care providers, the patient chose to continue the fruitarian diet.

At the follow-up visit in April, blood glucose control was adequate according to CGM metrics, although glucose time-above-range had increased compared to the previous period (Table 1). Daily glucose variability had increased (37%), as also evidenced by the CGM profile (Figure 1D). Basal/bolus proportion was similar to that in August 2020 (47% bolus and 53% basal). As compared to November 2020, he had reintroduced raw protein-rich vegetables (artichokes, mushrooms, carrots, and zucchini) with no major changes in the mean dietary composition, except for reduced intake of fructose and monounsaturated fatty acids (Table 2). However, he reported occasional episodes of uncontrolled behavior, when he was consuming different foods, mainly pizza and bread, in considerable amounts. He did not report changes in body weight and physical activity. Plasma triglyceride and liver enzymes concentrations were within normal ranges (Table 1). An increased plasma potassium concentration was detected (6 mmol/L).

Healthcare providers cautioned the patient on the harmful effects of this diet on his long-term health (i.e., increased glucose variability, liver abnormalities, and hyperkaliemia).

The patient was provided a dietary plan to switch to a more balanced diet, suggesting to increase the intake of plant proteins and fats by favoring the consumption of legumes, sprouted grains, and extra-virgin olive oil (6). Moreover, the patient was asked to reduce the amount of K-rich foods (i.e., banana, dates) and total fruits to standard servings (400–500 g/day), preferring whole fruits over juices because of their higher fiber content and lower glycemic index (7). Nevertheless, the patient expressed his will to continue the fruitarian diet mainly because of its environmental and social implications.

Therefore, healthcare providers planned a strict follow-up to monitor circulating liver enzymes and electrolyte concentrations, liver fat content, and potential vitamin and mineral deficiencies.

## Discussion

The fruitarian diet is based on fruits, nuts, and seeds. Vegetables that are classified botanically as fruits (i.e., avocado and tomatoes) are included, while other vegetables, namely, grains and beans, and animal products are excluded (6). Concerns about the fruitarian diet have focused mainly on its high fructose content. Indeed, high fructose intake may increase triglyceride concentrations by promoting hepatic lipogenesis, and several epidemiological studies have suggested an association between average daily fructose intake and liver steatosis (8, 9). Evidence from randomized controlled clinical trials is not conclusive, although some studies reported increased liver fat accumulation after fructose consumption in healthy individuals (10) or people with type 2 diabetes (11). To the best of our knowledge, no studies evaluating the effect of fructose consumption on liver fat in individuals with type 1 diabetes are currently available.

Other potential major flaws related to the fruitarian diet refer to poor diet quality and nutritional deficiencies, particularly in terms of protein, omega-3 fatty acids (EPA and DHA), and micronutrients (i.e., vitamin B12, iron, and zinc) (6).

This case report shows that a short-term fruitarian diet increases plasma triglyceride and liver enzymes concentrations, particularly in the first 3 months, with no significant effects on liver fat accumulation evident on ultrasound evaluation. Unlike processed and refined foods with simple sugars, fruits contain several bioactive compounds with antioxidant activity (vitamins, minerals, fiber, polyphenols, and other phytochemicals). These compounds might reduce oxidative stress and inflammation that are major contributors to liver steatosis.

Plasma triglyceride and liver enzymes tended to reduce after 6 months. This might be related to lower adherence to the strict fruitarian diet, as also stated by the patient.

As for blood glucose control, it has been shown that fructose is a slow digestible carbohydrate that induces a lower postprandial blood glucose response than glucose in individuals with type 1 diabetes (12, 13). This could explain the reduction in glucose variability in the first 3 months of the fruitarian diet. Of note, the patient experienced more user-friendliness and feasibility in tracking the grams of carbohydrate since he had gone fruitarian. Indeed, his habitual meal plan (Supplementary Table 1) does not contain mixed meals that could require advanced knowledge of foodstuff composition to adjust insulin doses to compensate for delayed postprandial glycemic excursions (1). Therefore, optimal carbohydrate counting may have contributed to the achievement of optimal glycemic control with reduced glucose variability.

On the other hand, as stated by the patient, the fruitarian diet is highly restrictive (i.e., limited food options, difficulty in attending social events), and it affected his meal regularity, resulting in occasional loss of control with high consumption of highly gratifying comfort foods. This may have contributed to his worsened blood glucose control in the last period, in addition to the reported high intake of orange juice (Supplementary Table 1) that has recently been demonstrated to negatively affect blood glucose control in a cohort of individuals with type 1 diabetes (14).

Another reason of concern was the increased plasma potassium (K) concentration observed after 6 months on the fruitarian diet that is in line with the fact that daily dietary K intake reported by the patient was three-fold higher than the recommended one (3.9 g/day). Previous studies have shown increased circulating K concentrations with no changes in renal function after short-term oral K supplementation (15).

To the best of our knowledge, there is no evidence on short- or long-term effects of the fruitarian diet in patients with type 1 diabetes. Nevertheless, according to the evidence existing on high-glycemic index and sugars, we would at present strongly discourage the use of the fruitarian diet for patients with diabetes. Further experimental clinical research is required on the usefulness and safety of extreme plant-based diets.

In conclusion, this case emphasizes the importance of close dietary counseling in the follow-up of patients with type 1 diabetes to ensure personalized medical nutrition therapy, in accordance with the current guidelines. This may allow for the correction of early alterations, not recognizable based only on blood glucose control, before incurring harmful consequences. In the era of social media, patients are exposed to fashionable dietary trends and ready-to-use information that might endanger their health. At the present state of knowledge, the fruitarian diet is not recommended for patients with diabetes.

## Data Availability Statement

The raw data supporting the conclusions of this article will be made available by the authors, without undue reservation.

## Ethics Statement

The studies involving human participants were reviewed and approved by Ethics Committee Federico II. The patients/participants provided their written informed consent to participate in this study. Written informed consent was obtained from the individual(s) for the publication of any potentially identifiable images or data included in this article.

## Author Contributions

CV and LB wrote the report and analyzed the data. GD collected the data and contributed to commentary. AR and GA provided relevant intellectual contributions to the development of the report. GA is the guarantor of this study and as such, had full access to all the patient data and takes responsibility for the integrity and accuracy of the case presentation. All authors revised the manuscript and gave final approval of the version to be submitted for publication.

## Conflict of Interest

The authors declare that the research was conducted in the absence of any commercial or financial relationships that could be construed as a potential conflict of interest.

## Publisher's Note

All claims expressed in this article are solely those of the authors and do not necessarily represent those of their affiliated organizations, or those of the publisher, the editors and the reviewers. Any product that may be evaluated in this article, or claim that may be made by its manufacturer, is not guaranteed or endorsed by the publisher.

## Supplementary Material

Supplementary Table 1 Examples of the daily menu as reported in the 7-day food records.

## References

[1] American Diabetes Association. Lifestyle management: standards of medical care in diabetes. Diabetes Care. (2019) 42(Suppl. 1):S46–60. 10.2337/dc19-S005 30559231

[2] Salas-Salvadó J Becerra-Tomás N Papandreou C Bulló M. Dietary patterns emphasizing the consumption of plant foods in the management of type 2 diabetes: a narrative review. Adv Nutr. (2019) 10:S320–31. 10.1093/advances/nmy102 31728494 PMC6855981

[3] Bolla AM Caretto A Laurenzi A Scavini M Piemonti L. Low-carb and ketogenic diets in type 1 and type 2 diabetes. Nutrients.(2019) 11:962. 10.3390/nu11050962 31035514 PMC6566854

[4] Cosentino F Grant PJ Aboyans V Bailey CJ Ceriello A Delgado V. 2019 ESC Guidelines on diabetes, pre-diabetes, and cardiovascular diseases developed in collaboration with the EASD. Eur Heart J. (2020) 41:255–323. 10.1093/eurheartj/ehz486 31497854

[5] Battelino T Danne T Bergenstal RM Amiel SA Beck R Biester T. Clinical target for continuous glucose monitoring data interpretation: recommendations from the international consensus on time in range. Diabetes Care. (2019) 42:1593–603. 10.2337/dci19-0028 31177185 PMC6973648

[6] Melina V Craig W Levin S. Position of the academy of nutrition and dietetics: vegetarian diets. J AcadNutr Diet. (2016) 116:1970–80. 10.1016/j.jand.2016.09.025 27886704

[7] Locke A Schneiderhan J Zick SM. Diets for health: goals and guidelines. Am Fam Physician. (2018) 97:721–8. 30215930

[8] Ter Horst KW Serlie MJ. Fructose consumption, lipogenesis, and non-alcoholic fatty liver disease. Nutrients. (2017) 9:981. 10.3390/nu9090981 28878197 PMC5622741

[9] Della Pepa G Vetrani C Lombardi G Bozzetto L Annuzzi G Rivellese AA. Isocaloric dietary changes and non-alcoholic fatty liver disease in high cardiometabolic risk individuals. Nutrients. (2017) 9:1065. 10.3390/nu9101065 28954437 PMC5691682

[10] Schwarz JM Noworolski SM Wen MJ Dyachenko A Prior JL Weinberg ME. Effect of a high-fructose weight-maintaining diet on lipogenesis and liver fat. J Clin Endocrinol Metab. (2015) 100:2434–42. 10.1210/jc.2014-3678 25825943 PMC4454806

[11] Taskinen MR Söderlund S Bogl LH Hakkarainen A Matikainen N Pietiläinen KH. Adverse effects of fructose on cardiometabolic risk factors and hepatic lipid metabolism in subjects with abdominal obesity. J InternMed. (2017) 282:187–201. 10.1111/joim.12632 28548281

[12] Souto DL Lima ÉDS Dantas JR Zajdenverg L Rodacki M Rosado EL. Postprandial metabolic effects of fructose and glucose in type 1 diabetes patients: a pilot randomized crossover clinical trial. Arch Endocrinol Metab. (2019) 63:376–84. 10.20945/2359-3997000000148 31365624 PMC10528643

[13] Dos Santos Lima É Souto DL Rodacki M Pereira JRD Zajdenverg L Rosado EL. Metabolic and appetite effects of fructose and glucose in subjects with type 1 diabetes: a randomized crossover clinical trial. Curr Diabetes Rev. (2021) 17:e113020188536. 10.2174/1573399816666201201092334 33261542

[14] Basu A Alman AC Snell-Bergeon JK. Associations of dietary patterns and nutrients with glycated hemoglobin in participants with and without type 1 diabetes. Nutrients. (2021) 13:1035. 10.3390/nu13031035 33806867 PMC8004940

[15] Cappuccio FP Buchanan LA Ji C Siani A Miller MA. Systematic review and meta-analysis of randomised controlled trials on the effects of potassium supplements on serum potassium and creatinine. BMJ Open.(2016) 6:e011716. 10.1136/bmjopen-2016-011716 27566636 PMC5013341

