# Supplementary material for: Fruitarian Diet and Blood Glucose Control in Type 1 Diabetes: A Case Report
Source: Front Nutr. 2022 Feb 22;9:752832. doi: 10.3389/fnut.2022.752832 (PMC8904176; doi:10.3389/fnut.2022.752832)
Supplement: Supplementary Table 1 — Examples of the daily menu as reported in the 7-day food records. [file Table_1.DOCX]

| **Supplementary table 1.** Examples of daily menu as reported in the 7-day food records. | |
| --- | --- |
| **November 2020** | **April 2021** |
| Lunch | |
| Tangerine 1000 g  Avocado 110 g | Banana 1000 g  Berry mix 150 g  Orange juice 1 Liter |
| Dinner | |
| Fresh dates 400 g  Banana 600 g | Apple 300 g  Artichokes 200 g |
